# Atom-Economic Synthesis and Morphology-Dependent Marine Biodegradation Behavior of 2‑Pyrone-4,6-Dicarboxylic Acid–Based Polyesteramide

**DOI:** 10.1021/polymscitech.6c00037

**Published:** 2026-05-12

**Authors:** Yijie Jin, Wei Xin Tan, Manjusha Joshi, Yasutaka Kuwahara, Shota Michida, Atsushi Isobe, Manabu Tanaka, Takuya Katashima, Masaya Fujita, Takuma Araki, Yuzo Suzuki, Yuichiro Otsuka, Naofumi Kamimura, Eiji Masai, Tsuyoshi Michinobu

**Affiliations:** † Department of Materials Science and Engineering, 13290 Institute of Science Tokyo, 2-12-1 Ookayama, Meguro-ku, Tokyo, 152-8552, Japan; ‡ School of Materials Science & Engineering, 54761 Nanyang Technological University, Singapore 639798, Singapore; § Department of Applied Chemistry, Faculty of Urban Environmental Sciences, 12944 Tokyo Metropolitan University, 1-1 Minami-osawa, Hachioji, Tokyo 192-0397, Japan; ∥ Department of Bioengineering, Graduate School of Engineering, 13143 The University of Tokyo, Bunkyo, Tokyo 113-8656, Japan; ⊥ Department of Materials Science and Bioengineering, 52756 Nagaoka University of Technology, Nagaoka 940-2188, Japan; # Department of Forest Resource Chemistry, Forestry and Forest Products Research Institute, Tsukuba 305-8687, Japan

**Keywords:** lignin, polyesteramide, marine biodegradation, morphology, ring-opening polyaddition, biomass-based polymer

## Abstract

2-Pyrone-4,6-dicarboxylic acid (PDC) is a lignin-derived biometabolic intermediate that serves as a versatile monomer for the development of sustainable polymers. In the present study, a bio-based polyesteramide, P­(PDC-BiOx), was successfully synthesized via the atom-economical ring-opening polyaddition of PDC and 2,2′-bis­(2-oxazoline). The thermal, crystalline, and mechanical properties of the resulting polymer were fully characterized, revealing a completely amorphous backbone with a fracture stress of 10.34 MPa. The marine biodegradability behavior was investigated under both surface and deep seawater conditions, demonstrating a clear correlation between material morphology and biodegradation rate. The experimental results demonstrated that, in surface seawater, the electrosprayed microparticles degraded more rapidly than the hot-pressed film, although both eventually reached a comparable extent of degradation. In comparison, in deep seawater, the electrosprayed microparticles achieved approximately 30% biodegradation within 45 days, which was higher than that of the hot-pressed film. Overall, PDC polyesteramides are promising biomass-derived polymers with straightforward accessibility and potential marine biodegradability.

## Graphical abstract

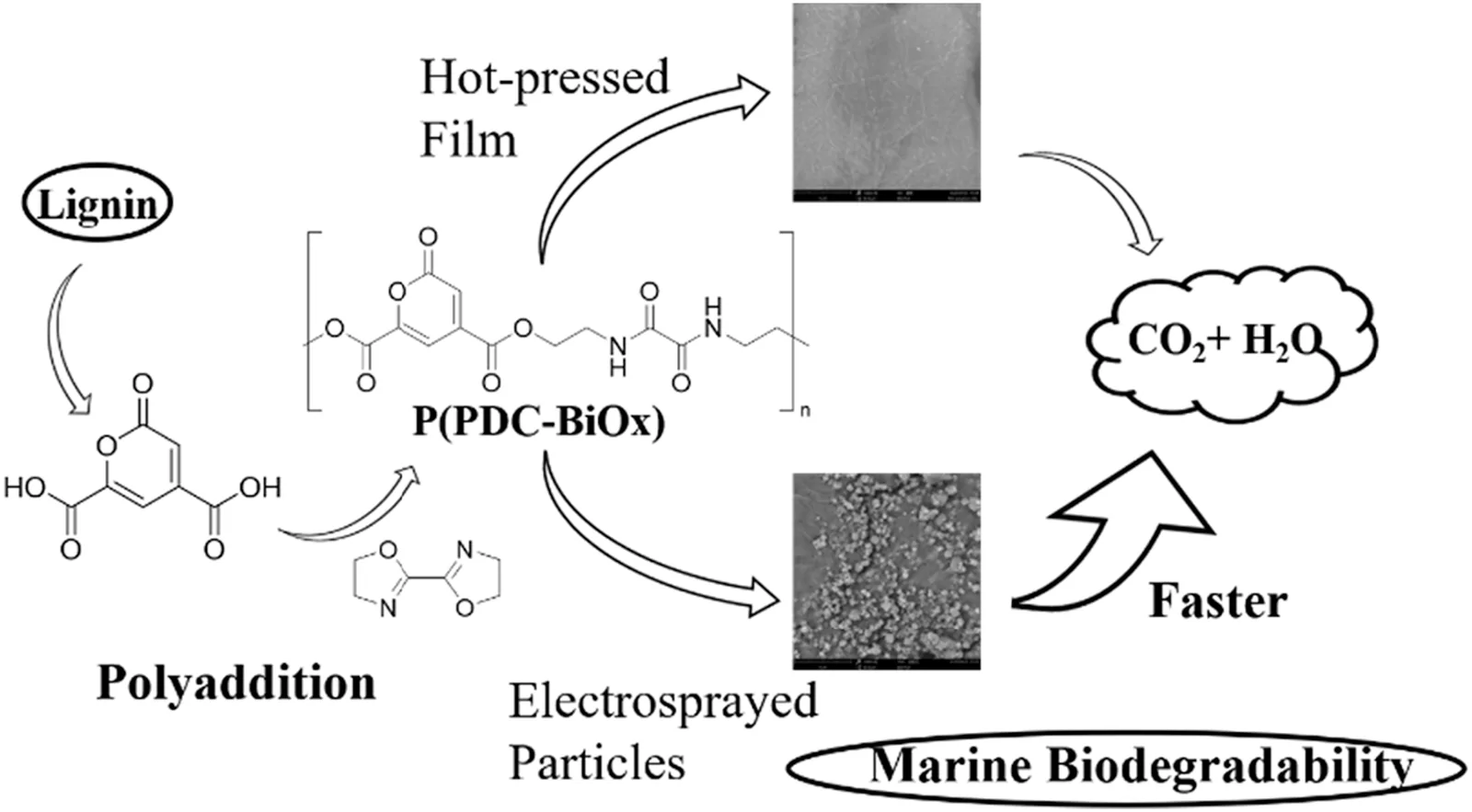

## 1 Introduction

The urgent need to transition toward a sustainable, carbon-neutral future has driven the development of bio-based renewable resources as viable alternatives to petroleum-based plastics.^1–8^ This trend has garnered widespread academic interest in the development of high-performance biomaterials, aiming to reduce dependence on fossil resources through a closed-loop carbon cycle.^9–12^ Among the diverse range of bio-based materials being explored, lignin is a particularly promising candidate due to its ubiquity and abundance in nature.^13–17^ As the largest naturally occurring renewable aromatic polymer, lignin possesses a complex three-dimensional network structure, rendering it an attractive feedstock with significant potential for high thermal stability and mechanical strength.^18,19^ However, the structural complexity and heterogeneity of lignin also present major technical challenges for its valorization, as traditional physical or chemical depolymerization methods often struggle to precisely control the molecular structure of the resulting products.

To overcome these limitations, a promising strategy has been the use of microorganisms to selectively depolymerize lignin and convert it into valuable, small molecular compounds.^20–23^ In recent advances in metabolic engineering, engineered *Pseudomonas putida* strains have been successfully utilized to convert lignin-derived aromatic molecules into 2-pyrone-4,6-dicarboxylic acid (PDC), a biometabolic intermediate, on a large scale.^24^ From a structural perspective, PDC consists of a polar pseudo-aromatic pyrone ring and two carboxyl groups, making it an ideal multifunctional monomer for the synthesis of various high-performance polymers that often exhibit unique physicochemical properties.^25–30^ In our previous studies, various PDC-based polyesters synthesized via polycondensation demonstrated excellent environmental friendliness and the potential for full microbial biodegradation in freshwater environments.^31,32^ However, it is generally known that polyamides are more mechanically tough than polyesters. PDC-based polyamides have been reported, but their synthesis requires specific conditions because the pyrone ring is usually sensitive to amine molecules.^27,33,34^ In addition, polyamides are generally less biodegradable than polyesters. Therefore, it is significant to control the balance between the mechanical strength and biodegradability of biomass-based polymers. Polyesteramides can be synthesized by the ring-opening reaction of bis­(2-oxazoline)­s with dicarboxylic acids,^35^ but it has never been applied to PDC. To further expand the functional boundaries of lignin-derived materials, particularly for the development of novel frameworks with higher chemical stability and controllable degradation characteristics, exploring a new synthetic pathway for PDC is of great importance.

In the present study, we report the synthesis and characterization of a novel PDC-based linear polyesteramide, P­(PDC-BiOx), prepared via the ring-opening polyaddition between PDC and 2,2′-bis­(2-oxazoline). The polymer was soluble in polar organic solvents, such as *N*,*N*-dimethylformamide (DMF). Compared to traditional polycondensation, the advantage of this synthetic strategy lies in the absence of byproducts during polymerization, aligning with the principles of sustainable chemistry. We investigated the thermal and mechanical properties of the PDC polyesteramide and evaluated their biodegradation behavior in a simulated marine environment. Given the complexity of marine ecosystems, the microscopic morphology of the materials may significantly influence degradation efficiency. In this context, we prepared two different samples of PDC polyesteramide: hot-press films and electrosprayed microparticles. By comparing these samples, we demonstrate how the physical form of the material affects the degradation kinetics and extent under different marine conditions. The findings in this study provide a strategic approach for tuning the degradation behavior of PDC-based polymers by controlling material morphology and surface area.

## 2 Materials and Methods

### 2.1 Materials

2-Pyrone-4,6-dicarboxylic acid (PDC) was prepared from vanillic acid using an engineered *P. putida* strain, as reported previously.^24^ 2,2′-Bis­(2-oxazoline) was purchased from Tokyo Kasei Kogyo Co., Ltd. (Tokyo, Japan). DMF was purchased from Kanto Chemical Co., Inc. (Tokyo, Japan).

Natural seawater samples were collected from the Water Intake Supply Facility of the Suruga Bay Deep Seawater Fisheries Utilization Facility (Yaizu, Shizuoka, Japan), utilizing both surface seawater and deep seawater collected at a depth of 397 m. Prior to use, coarse particles were removed from the seawater, and the seawater was pre-conditioned to reduce the total organic carbon (TOC) content, referring to ISO 23977-2:2020. For the preparation of the biodegradation test medium, ammonium chloride (NH_4_Cl) and anhydrous potassium dihydrogen phosphate (KH_2_PO_4_) were purchased from Kanto Chemical Co., Inc. (Tokyo, Japan). Cellulose (microcrystalline) was purchased from Kanto Chemical Co., Inc. (Tokyo, Japan). Ashless cellulose filters were purchased from ADVANTEC (Tokyo, Japan). Poly­(3-hydroxybutyrate-*co*-3-hydroxyvalerate) (PHBV, Y1000) powder was used. All reagents were used without further purification.

### 2.2 Measurements

Nuclear magnetic resonance (^1^H-NMR) spectra were recorded on a JEOL (Tokyo, Japan) model 400YH spectrometer at 20 °C. All chemical shifts are reported in parts per million downfield from SiMe_4_, using the solvent’s residual signal as an internal reference. Fourier transform infrared spectra (FT-IR) were recorded on a JASCO (Tokyo, Japan) FT/IR-4200 spectrometer. Gel permeation chromatography (GPC) was measured at 40 °C using a JASCO HSS-1500 system with a refractive index (RI) detector and two Shodex GPC KF-803 columns (8.0 mm I.D. × 300 mm L). DMF containing LiBr (5 mM) was used as the eluent at the flow rate of 1.0 mL min^–1^ at 40 °C. The molecular weights were calculated based on a calibration curve generated using poly­(methyl methacrylate) (PMMA) standards. Thermogravimetric analysis (TGA) and differential scanning calorimetry (DSC) measurements were recorded under a nitrogen flow on a Rigaku (Tokyo, Japan) Thermoplus TG8120 and DSC8230, respectively, at a scanning rate of 10 °C min^–1^. Scanning electron microscopy (SEM) images of the pristine films and microparticles were acquired using a Phenom ProX desktop SEM (PW-100-517, Phenom-World BV). For the characterization of the biodegraded specimens, a field-emission SEM (JSM-7500F, JEOL Ltd., Tokyo, Japan) was employed to observe the surface morphological changes. Atomic force microscopy (AFM) measurements were performed at room temperature in air using a NanoWizard Sense system (Bruker Nano GmbH, Berlin, Germany) equipped with an OMCL-AC240TS cantilever (Olympus, Tokyo, Japan). X-ray diffraction (XRD) measurements were performed using a PANalytical (Almelo, The Netherlands) X-ray diffraction (XRD) instrument X′Pert MPD at the Core Facility Center, Institute of Science Tokyo.

Nitrogen adsorption–desorption isotherms were measured at 77 K using a BELSORP-Max (Microtrac-BEL Corp., Japan) automatic gas adsorption analyzer. The specific surface area was calculated using the Brunauer–Emmett–Teller (BET) method. Pore size distributions were analyzed using the Barrett-Joyner-Halenda (BJH) method for mesopores, and the Horvath-Kawazoe (HK) and Saito-Foley (SF) methods for micropores.

### 2.3 Electrosprayed Sample Preparation

Electrosprayed microparticles were prepared using an NES-101A system (MECC Co. Ltd., Fukuoka, Japan). The polymer solution dissolved in anhydrous DMF was sprayed through an 18G syringe needle. The process was conducted at an applied voltage of 18 kV with a flow rate of 0.5 mL h^–1^, and the tip-to-collector distance was set to 12.5 cm. The resulting particles were collected from the substrate for subsequent tests.

### 2.4 Mechanical Properties

PDC-based polyesteramide films were prepared by hot pressing at 10 MPa and 150 °C, followed by curing under vacuum for 10 min. To control the film thickness, a 0.30 mm thick Teflon spacer was used to keep the thickness of the films at 0.30 ± 0.05 mm. After slowly cooling to room temperature, the films were cut into dumbbell-shaped specimens using an SDL-100 (Japan) SD-type lever-controlled sample cutter. The specimens were then subjected to stress-strain (S–S) analysis at 23 ± 2 °C using a Shimadzu Co. (Tokyo, Japan) autograph AGS-10kNX STD.

### 2.5 Rheological Measurements

Macroscopic rheological measurements were carried out using a stress-controlled rheometer (MCR 301, Anton Paar, Graz, Austria) equipped with a parallel-plate geometry (50 mm diameter, 1 mm gap). The poly­(vinyl alcohol) (PVA) solution was prepared by dissolving PVA in deionized water at 8 wt %. P­(PDC-BiOx) was dissolved in DMF at 30 wt %. Rheological measurements of the P­(PDC-BiOx) solutions were performed at 2, 30, and 130 h after sample preparation to capture the time-dependent evolution of their viscoelastic properties.

### 2.5.1 Dynamic Viscoelastic Measurement

Storage and loss moduli were evaluated by the dynamic viscoelastic measurement in the linear strain region (strain: 0.1–0.3). The frequency sweeps were performed between 0.1 and 100 rad s^–1^ at 25 °C. Complex viscosity |η*| was calculated by

1 $$\left|\eta^{*}\right|=\frac{\left|G^{*}\right|}{\omega }=\frac{\sqrt{G^{\prime 2}+G^{\prime \prime 2}}}{\omega }$$

where ω is the angular frequency, and *G*′ and *G*″ are the storage modulus and loss moduli, respectively.

#### 2.5.2 Stress Relaxation

Stress relaxation test was performed in the linear strain region (strain: 0.1–0.3). The stress relaxation data were transformed into dynamic moduli data by time–frequency transformation based on linear viscoelastic theory.

#### 2.5.3 Steady-Flow Measurement

For steady-flow measurements, constant shear rates γ̇ were applied for 2 min each in the linear-to-nonlinear regions (γ̇ = 0.1–3000 s^–1^). Measurements were performed with a 5 min interval, increasing from low to high shear rates.

### 2.6 Biodegradation Measurements

Biochemical oxygen demand (BOD) tests were performed to evaluate the biodegradability of the PDC-based polyesteramide in two different forms (films and electrosprayed microparticles) in a simulated marine environment using an OxiTop IS-6 (WTW GmbH, Weilheim in Oberbayern, Germany) referring to the ISO 23977-2:2020 standard. To support microbial activity and prevent nutrient deficiency during the test, the natural seawater was supplemented with inorganic salts: KH_2_PO_4_ (0.1 g L^–1^) and NH_4_Cl (0.05 g L^–1^).

Briefly, the biodegradation tests were performed in the dark, in closed 250 mL vessels, and incubated at 25 °C. Approximately 90 mL of the nutrient-supplemented seawater was added to each vessel containing 10 mg of the test material. Microcrystalline cellulose and ashless cellulose filters were used as reference materials for the microparticle and film samples, respectively, to validate the activity of the microbial population. Vessels containing only seawater without test material served as blanks. Each group (test samples, references, and blanks) consisted of three replicate vessels. Evolved CO_2_ was trapped by sodium hydroxide (350 mg) inside the vessels. The oxygen consumption in the vessel was determined from the pressure changes in the closed vessels. The biodegradability was estimated using the following equation

2 $$\mathrm{biodegradability}\left(\%\right)=\frac{{\mathrm{BOD}}_{\mathrm{sample}}-{\mathrm{BOD}}_{\mathrm{blank}}}{\mathrm{ThOD}}\times 100$$

where BOD_sample_ (mg L^–1^) represents the amount of oxygen consumption from a vessel and test material at time *t*, BOD_blank_ (mg L^–1^) is the average amount of oxygen consumption of the blank at time *t*, and ThOD (mg L^–1^) is the maximum amount of oxygen consumption that could be theoretically consumed by the test material based on the amount of added carbon.

For the preparation of test materials, the film samples were cut into small pieces (approximately 2 mm × 2 mm) in accordance with ISO 23977-2 recommendations. The electrosprayed microparticles were used as prepared, as their dimensions were inherently below the 250 μm threshold.

### 2.7 Synthesis

#### 2.7.1 P­(PDC-BiOx)

2-Pyrone-4,6-dicarboxylic acid (PDC, 1.00 g, 5.43 mmol) and 2,2’-bis­(2-oxazoline) (0.76 g, 5.43 mmol) were taken in a round-bottom flask containing anhydrous DMF (2.0 mL) under a nitrogen atmosphere. The reaction mixture was stirred at 120 °C for 3 h. The mixture was then cooled to room temperature and precipitated by dropwise addition into methanol (50 mL). The resulting solid was collected by filtration and dried under vacuum at 40 °C to yield P­(PDC-BiOx) (1.31 g, 74.4%) as an off-white solid. ^1^H NMR (399 MHz, DMSO-*d*_6_, δ, ppm): 9.11 (s, 1H), 9.01 (s, 1H), 7.28 (s, 1H), 7.14 (s, 1H), 4.34–4.24 (m, 4H), 3.50–3.43 (m, 4H). IR (neat): *ν* = 3320 (w), 1731 (s), 1665 (s), 1515 (m), 1456 (w), 1397 (w), 1331 (w), 1247 (s), 1173 (m), 1113 (w), 1019 (w), 877 (w), 761 (m), 711 (w) cm^–1^.

## 3 Results and Discussion

### 3.1 Synthesis and Characterization

The bio-based polyesteramide, P­(PDC-BiOx), was successfully synthesized via ring-opening polyaddition between the carboxylic acid groups of PDC and the oxazoline rings of 2,2′-bis­(2-oxazoline) (Scheme 1). This synthetic route follows an atom-economic polyaddition mechanism and proceeds without the elimination of small molecule by-products, thereby minimizing environmental impact. Mechanistically, the reaction is initiated by protonation of the nitrogen atom in the oxazoline ring by the carboxylic acid group of PDC, leading to the formation of an oxazolinium cation and a carboxylate anion. The carboxylate anion subsequently undergoes a nucleophilic attack at the 5-position carbon of the oxazolinium ring. This ring-opening process cleaves the carbon–oxygen bond, directly generating both ester and amide linkages that constitute the polymer backbone (Scheme S1).

**Scheme 1:**
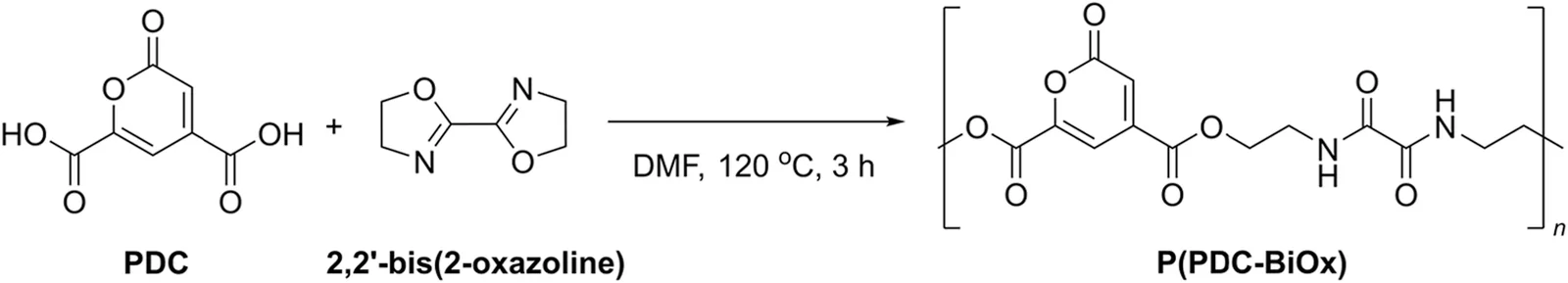
Synthesis of P­(PDC-BiOx) via Ring-Opening Polyaddition of PDC and 2,2′-bis­(2-Oxazoline)

To optimize the polymerization conditions, the reaction was investigated at temperatures ranging from 80 to 150 °C (Table S1). The results indicate that the reaction temperature significantly influenced the molecular weight and dispersity of the polymers. At 80 °C, only oligomers with a relatively low *M_n_* of 4300 g mol^–1^ were obtained (Figure S1), which is attributed to the low reactivity of the monomers at this temperature. Increasing the temperature to 100 °C and further to 120 °C promoted chain growth, with the reaction at 120 °C yielding the highest *M_n_* and the reasonable PDI of 11,700 g mol^–1^ and 1.74, respectively (Figure S1). A further increase in temperature to 150 °C resulted in the formation of an insoluble, brown, hard precipitate. It is speculated that this phenomenon might be caused by thermal crosslinking or decomposition. Previous studies have demonstrated that the pseudo-aromatic pyrone ring of PDC is structurally labile and highly susceptible to ring-opening reactions at temperatures around 150 °C under reactive chemical conditions.^31^ In the context of our study, although the amide linkages are inherently formed during polymerization,^35^ it is plausible that the combination of these species and the elevated temperature could induce a nucleophilic attack on the sensitive PDC ring.^36^ Such a side reaction, if present, would lead to the destruction of the polymer backbone and explain the observed deep brown color. Consequently, the polymer synthesized at 120 °C was selected for further characterization and biodegradation studies.

The chemical structure of P­(PDC-BiOx) was characterized by ^1^H NMR spectroscopy (Figure 1a). The appearance of amide proton signals at δ = 9.11 and 9.01 ppm confirmed the formation of the ester–amide backbone. The aromatic protons of the PDC units were clearly observed at δ = 7.28 and 7.14 ppm. Additionally, the methylene protons derived from the ring-opened oxazoline appeared as multiplets at δ = 4.24–4.34 ppm and 3.43–3.50 ppm, confirming the successful synthesis of the target polymer. FT-IR analysis was performed to identify the functional groups of P­(PDC-BiOx) (Figure 1b). Three prominent peaks were observed at 1730, 1660, and 1520 cm^–1^, corresponding to the ester C=O stretching, the amide I band, and the amide II band, respectively. The appearance of a broad peak at 3320 cm^–1^ was assigned to the N–H stretching vibration, further confirming the amide formation. The C–O–C stretching vibrations between 1170 and 1250 cm^–1^ supported the formation of ester linkages. Notably, the characteristic signals corresponding to the oxazoline C=N stretching and the carboxylic acid O–H groups were absent in the product, confirming high monomer conversion and successful construction of the polyesteramide backbone.

**Figure 1:**
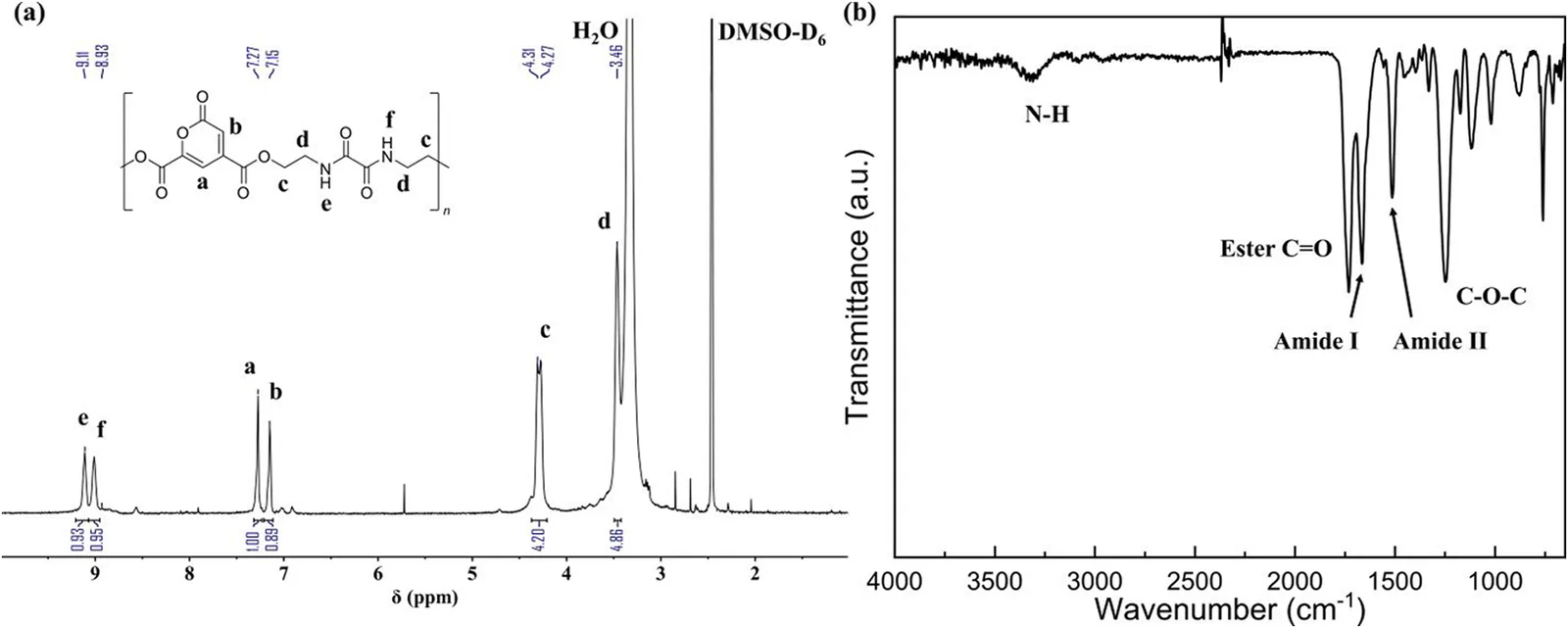
(a) ^1^H NMR spectrum of P­(PDC-BiOx) in DMSO-*d*_6_ at 20 °C and (b) FT-IR spectrum of P­(PDC-BiOx).

### 3.2 Thermal and Mechanical Properties

The thermal stability of P­(PDC-BiOx) was evaluated by TGA (Figure 2). The TGA result revealed that the polymer exhibits good thermal stability with a decomposition temperature at 5% weight loss of 224 °C. To investigate the physical state of the polymer solids, DSC and XRD analyses were performed. The glass transition temperature (*T*_g_) of P­(PDC-BiOx) was determined to be approximately 32 °C (Figure S2). The absence of endothermic melting peaks or exothermic crystallization peaks during the heating cycle indicates that the polymer possesses an amorphous character. Due to the unique pseudo-aromatic structure of the lignin-derived PDC monomer, directly comparable bio-based polyesteramides are rare in the literature. However, to contextualize its thermal behavior, P­(PDC-BiOx) was compared with previously reported PDC-based aliphatic polyesters.^32^ For instance, P­(PDC8), which contains an octamethylene flexible spacer, exhibits a 5% weight loss temperature of approximately 298 °C and a *T*_g_ of 44.1 °C. In comparison, P­(PDC-BiOx) displays a slightly lower *T*_g_ and a relatively lower thermal decomposition temperature. Nonetheless, this *T*_g_ value is slightly above room temperature, indicating a glassy state under ambient conditions, which is consistent with the brittle fracture behavior observed in the polymer films.

**Figure 2:**
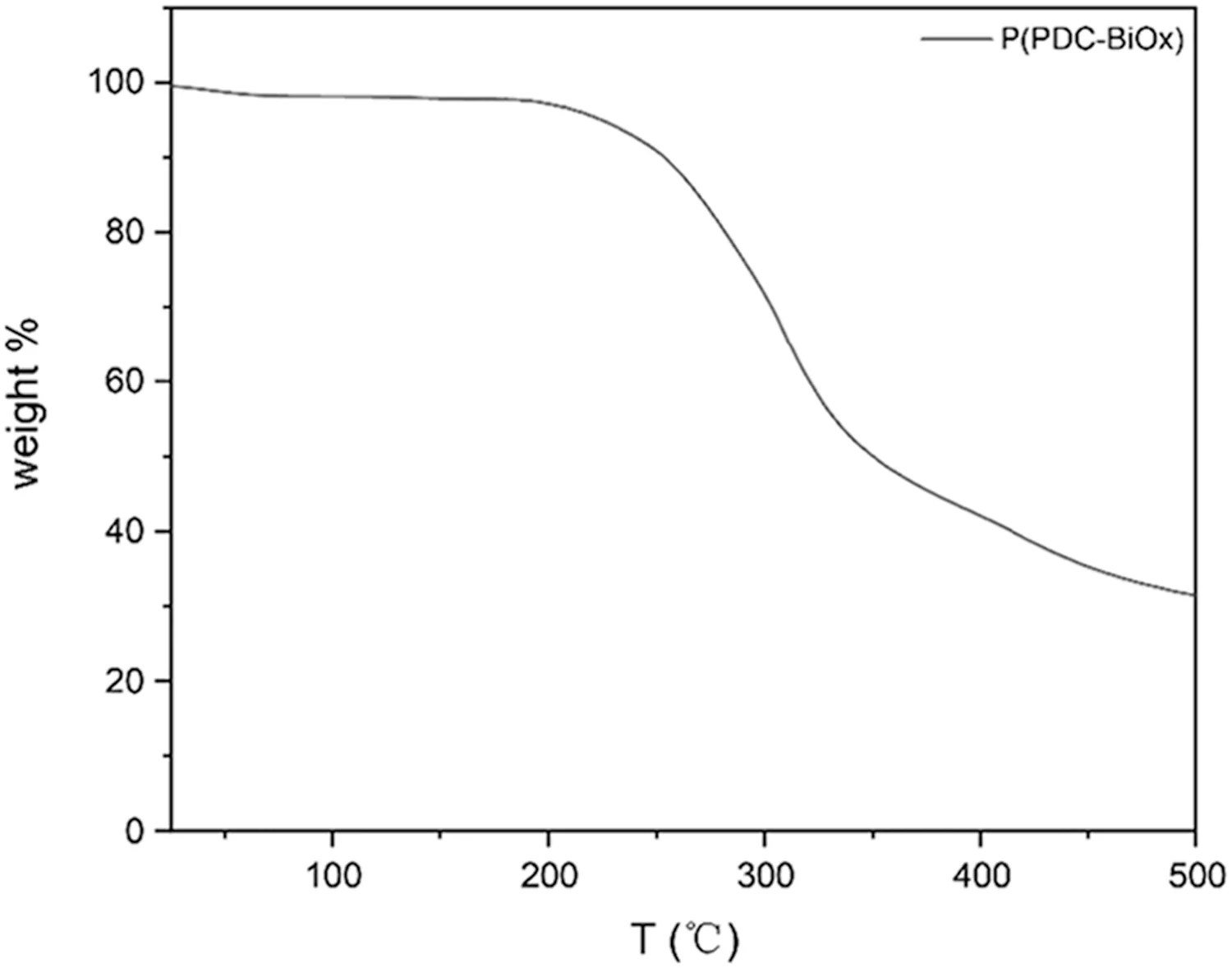
TGA curves of P­(PDC-BiOx) at the heating rate of 10 °C min^–1^ under flowing nitrogen.

The mechanical properties of P­(PDC-BiOx) were evaluated using hot-pressed films (Figure S3). Quantitative analysis of the stress-strain curve revealed that the polymer displayed a tensile strength of 10.34 MPa, a Young’s modulus of 618 MPa, and a low elongation at break of 1.90%. These specific values confirm a fundamentally brittle and fragile nature. This behavior is attributed to the rigid backbone structure consisting of heterocyclic PDC units and hydrogen-bonding amide groups with limited flexible segments,^32^ resulting in high stiffness but low toughness. To better contextualize these properties, P­(PDC-BiOx) can be compared with PDC-based aliphatic polyesters. Structurally, the spacer between PDC units in P­(PDC-BiOx) comprises a rigid oxamide core flanked by short ethylene segments. When compared to P­(PDC8), which possesses a fully flexible eight-carbon aliphatic spacer of similar main-chain repeat unit length and exhibits highly ductile behavior (fracture strain of 393%, Young’s modulus of 111 MPa), P­(PDC-BiOx) displays significantly higher stiffness and extreme brittleness. This striking contrast highlights that the rigid amide segments and strong intermolecular hydrogen bonding severely restrict chain mobility. Furthermore, while the direct polyester analog P­(PDC2), which contains only the short ethylene spacer, was too brittle to even form a free-standing film, P­(PDC-BiOx) gains sufficient cohesive strength from its amide hydrogen bonds to successfully form testable, albeit rigid, films. Although these properties may limit its applicability in high-load structural materials, its brittle nature is expected to facilitate physical fragmentation in marine environments, potentially accelerating macroscopic disintegration during the biodegradation process.

### 3.3 Morphology and Porosity Analysis

The morphology and surface area of polymeric materials significantly affect the biodegradability in nature. To study the morphological effect, two different samples were prepared from P­(PDC-BiOx). One is a hot-pressed film, in which the polymer chains are densely and tightly packed, resulting in a low effective surface area. The other is an electrospun sample, in which a high voltage is applied to a polymer solution and ultrafine fibers are usually generated via electrostatic forces, resulting in a high specific surface area. The hot-pressed film with a thickness of 0.30 mm was successfully prepared at 150 °C under a pressure of 10 MPa. In contrast, although electrospinning is typically a technique for producing microfibers, no filamentous products were obtained in this study despite extensive optimization of the processing conditions, including applied voltage between 15 and 30 kV, flow rate between 0.1 and 1.0 mL h^–1^, and tip-to-collector distance between 10 and 15 cm. Even though various combinations of the electrospinning parameters were investigated, only fine microparticles were formed. The reason for this result will be discussed in the following section based on rheological measurement of the P­(PDC-BiOx) solution. Therefore, in this study, we decided to use the electrosprayed microparticles as the second sample.

The surface morphology of the P­(PDC-BiOx) samples was investigated using SEM (Figure 3). The hot-pressed film exhibited a smooth, continuous, and dense surface (Figure 3a), consistent with the compact structure formed during hot pressing. In contrast, the electrosprayed sample displayed a completely different morphology consisting of discrete microparticles with diameters predominantly ranging from 0.5 to 2.0 μm (Figure 3b). To complement the SEM observations, atomic force microscopy (AFM) was employed to evaluate microscale topological changes in the hot-pressed film during degradation (Figure S4). The pristine film displayed a relatively continuous and smooth surface. Following the marine biodegradation, the surface exhibited a pronounced increase in roughness, characterized by deep crevices and numerous localized erosion pits. This dynamically roughened morphology increases the bio-accessible surface area, providing favorable sites for microbial adhesion and subsequent enzymatic attack.

**Figure 3:**
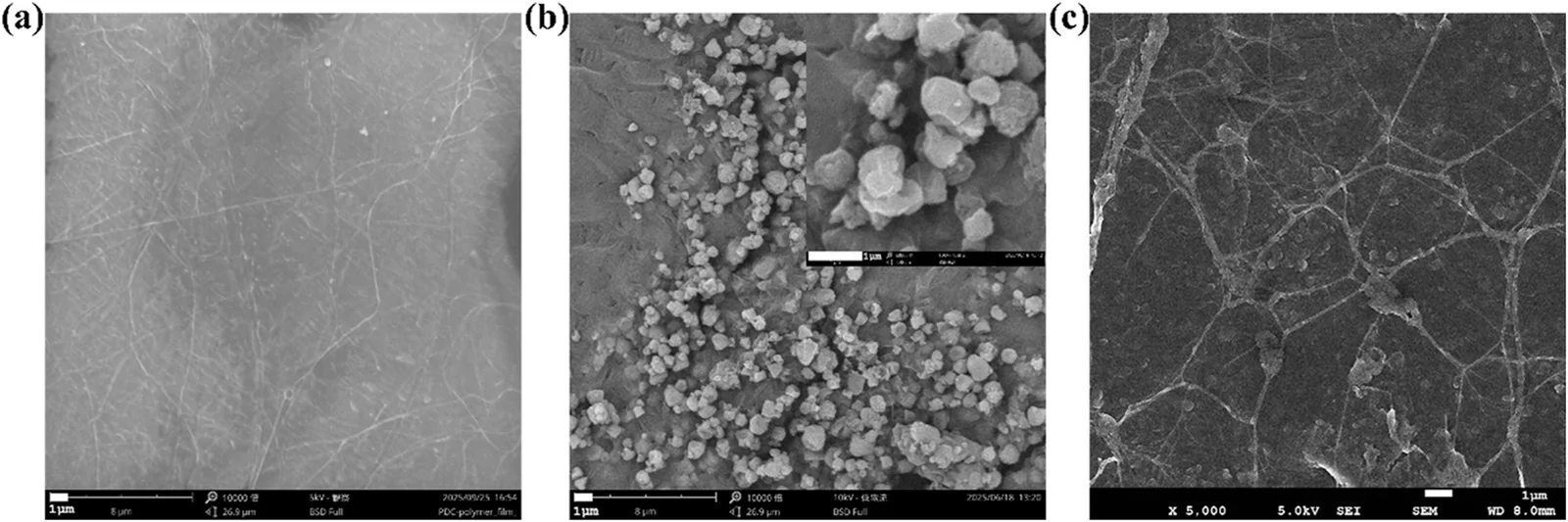
SEM images showing the morphological changes of P­(PDC-BiOx) for (a) the pristine hot-pressed film, (b) electrosprayed microparticles (the inset shows a higher magnification view), and (c) the hot-pressed film after 7 days of marine biodegradation. The white scale bars represent 1 μm.

XRD measurements confirmed the amorphous nature of both samples (Figure 4). Both the hot-pressed film and electrosprayed particles exhibited only broad amorphous halos without any sharp diffraction peaks, indicating that the polymer remains amorphous under these two specific processing conditions. The lack of crystallinity in P­(PDC-BiOx) can be attributed to the structural irregularity and steric hindrance of the PDC-based backbone, which prevents the organized packing of polymer chains. From the perspective of biodegradation, this amorphous nature is expected to facilitate moisture permeation and enzymatic attack, as it lacks crystalline regions that act as barriers to microbial erosion in natural environments.

**Figure 4:**
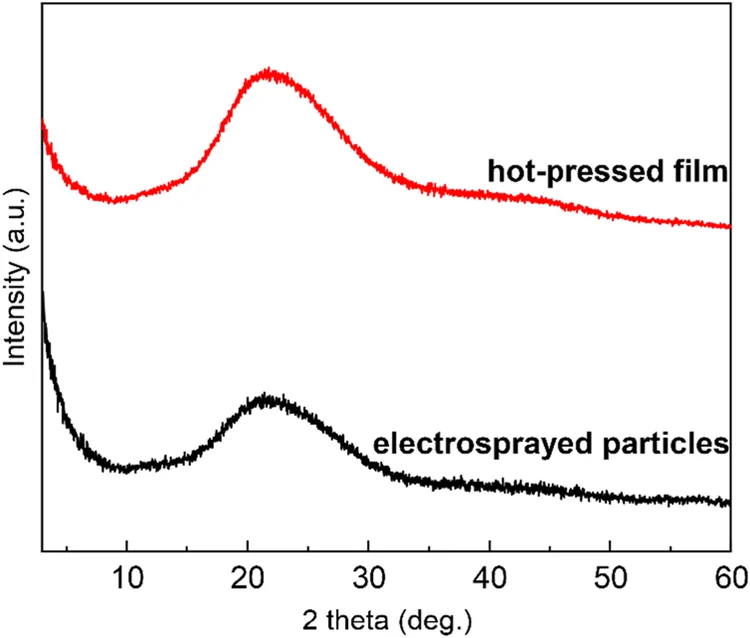
XRD patterns of P­(PDC-BiOx) hot-pressed film and electrosprayed particles.

To further characterize these morphological differences, N_2_ adsorption-desorption isotherms were measured. The BET analysis of the hot-pressed film was consistent with SEM observations, revealing a low specific surface area of ∼0.4 m^2^ g^–1^, whereas the electrosprayed microparticles exhibited a distinct Type II isotherm with an H3-type hysteresis loop (Figure S5). The sharp increase in adsorption at high relative pressures where *p*/*p*_0_ exceeds 0.9 is characteristic of inter-particle voids formed by the accumulation of microspheres. Accordingly, the specific surface area of the electrosprayed particles significantly increased to 6.14 m^2^ g^–1^, which is approximately 14 times higher than that of the hot-pressed film. The average pore diameter and total pore volume were determined to be 21.9 nm and 0.034 cm^3^ g^–1^, respectively. This substantial increase in the surface-to-volume ratio is expected to enhance the accessibility of seawater and microbial enzymes to the polymer chains, thereby promoting biodegradation.

### 3.4 Rheological Properties

Although electrospinning was originally intended to produce fibrous samples in order to maximize the surface-to-volume ratio, the process instead yielded microparticles. To elucidate the underlying mechanism of microparticle formation, the rheological properties of the P­(PDC-BiOx) solution in DMF were investigated. The angular-frequency dependence of the storage and loss moduli for the 30 wt % P­(PDC-BiOx) solution was measured (Figure 5). As a control, 8 wt % PVA aqueous solution was also measured under the same conditions. For the PVA solution, *G*″ exceeded *G*′ over the entire frequency range investigated, and *G*′ and *G*″ showed the scaling relationships that *G*′ ∼ ω^2^ and *G″* ∼ ω, indicating the terminal relaxation regime. A similar viscoelastic response, characterized by *G*″ > *G*′ and *G*″ ∼ ω, was also observed for the P­(PDC-BiOx) solution up to 30 h after dissolving this polymer in DMF.^37^ During this period, both *G*′ and *G*″ gradually increased with incubation time, reflecting the progressive formation of hydrogen bonds and the accompanying increase in the apparent molecular weight. At 130 h after preparation, the P­(PDC-BiOx) solution exhibited a pronounced elastic plateau of *G*′ within the intermediate frequency range, larger than *G*″. On the other hand, at lower frequencies, *G*″ exceeded *G*′, demonstrating that the system remained in a liquid state and had not yet reached a permanently crosslinked gel. These features suggest the formation of transient physical networks arising from the physical aggregation.

**Figure 5:**
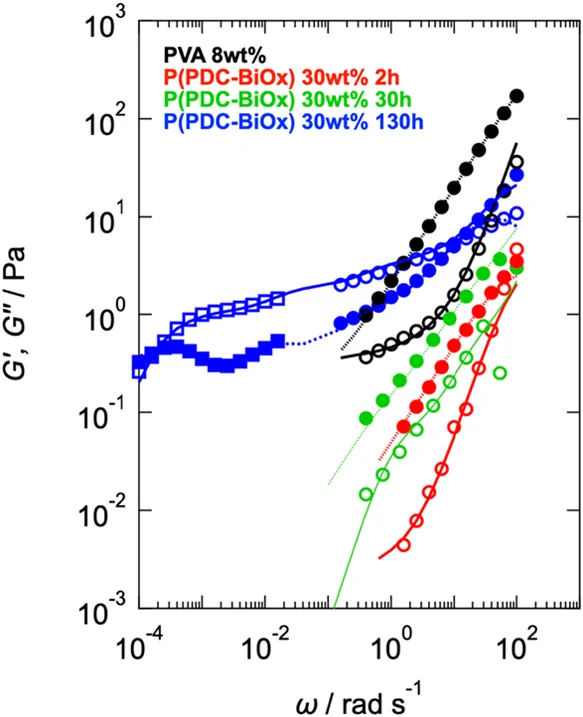
Angular frequency dependence of the dynamic moduli. Open and closed symbols represent the storage and loss moduli, respectively (circles: dynamic viscoelastic measurement; squares: data converted from stress relaxation tests). Dashed and solid lines represent the generalized Maxwellian model fits for the storage and loss moduli, respectively.

The steady-shear viscosity, *η*_st_, and the complex viscosity, |η*|, were compared as functions of shear rate and angular frequency, respectively (Figure 6). For the PVA solutions, *η*_st_ showed a plateau in the low shear rate and reduced in the high shear range, which is shear thinning. The shear rate dependence of *η*_st_ agrees with the angular frequency dependence of |η*| (Cox–Merz rule). The features are well known in the entangled linear polymer melts and solutions.^38^ On the other hand, for the P­(PDC-BiOx) solution up to 30 h, *η*_st_ was almost independent of shear rate (Newtonian behavior), suggesting that the apparent molar mass of P­(PDC-BiOx) is significantly low and the entanglement effect is negligible. The P­(PDC-BiOx) solution aged for 130 h exhibited clear non-Newtonian behavior, with *η*_st_ decreasing and reaching a plateau with increasing shear rate. In addition, while *η*_st_ and |η*| coincided at low shear rates and low frequencies, a pronounced deviation of the type *η*_st_ > |η*| emerged at higher shear rates, which is the breakdown of the Cox–Merz rule. Such an unusual Cox–Merz deviation has been reported for solutions of comb-shaped polystyrene, where the degree of deviation increases with increasing branch length.^39^ Computational studies by Snijkers and co-workers demonstrated that, for polymers with sufficiently long branches, the backbone begins to stretch at shear rates on the order of the inverse terminal relaxation time.^40^ This early onset of backbone stretching, often referred to as enhanced stretching, leads to a higher steady-shear viscosity compared to the complex viscosity measured under small-amplitude oscillatory shear, where the imposed deformation is too weak to induce significant backbone extension. By analogy, the observed deviation in the aged P­(PDC-BiOx) solution can be attributed to the slow, time-dependent physical aggregation of the polymer chains, forming transient physical networks via hydrogen bonding. It is worth noting that while minor evaporation of the DMF solvent over 130 h could slightly increase the overall polymer concentration, such a simple concentration effect cannot account for the severe breakdown of the Cox–Merz rule and the emergence of an elastic plateau. These drastic rheological transitions explicitly confirm that the formation of a physical hydrogen-bonded network, rather than solvent evaporation, is the dominant factor. Under oscillatory shear in the linear regime, these structures remain largely intact and contribute mainly to the elastic response, as evidenced by the appearance of a plateau in *G*′. Under steady shear, however, the same structures are stretched and partially rearranged, leading to an anomalous increase in *η*_st_ relative to |η*|. This interpretation is fully consistent with the viscoelastic signatures observed in Figure 5 and supports the conclusion that branching and transient network formation dominate the rheological evolution of the P­(PDC-BiOx) solution at long aging times.

**Figure 6:**
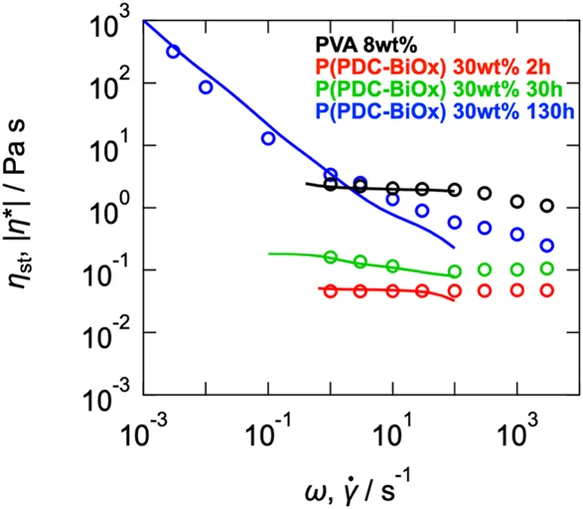
Cox–Merz applicability for PVA solution and P­(PDC-BiOx) solution. Steady shear viscosity (open circles) from steady flow experiments and complex viscosity (solid lines) from dynamic viscoelastic measurement plotted with respect to each other to determine the pertinence of the Cox–Merz rule at 25 °C.

The rheological results also provide insight into the contrasting electrospinnability of PVA and P­(PDC-BiOx) solutions. PVA solutions can be readily electrospun, which is consistent with their sufficiently high viscosity and pronounced shear-thinning behavior, both of which are essential for stabilizing the electrified jet against capillary breakup. In contrast, P­(PDC-BiOx) solutions shortly after preparation exhibit viscosities more than an order of magnitude lower than those of PVA solutions and behave as simple Newtonian liquids. Such low viscosity likely results in insufficient viscoelastic resistance to suppress jet instabilities, leading to electrospinning failure at early stages. Beyond rheology, this phenomenon is also strongly influenced by surface tension and the solvent evaporation rate. Specifically, the low degree of chain entanglement does not provide sufficient cohesive force to counterbalance surface tension, which promotes capillary breakup of the jet into discrete droplets to minimize interfacial area. This tendency is further amplified when DMF is used as the solvent. Owing to its high boiling point and slow evaporation rate, the jet remains fluid for an extended period during its transit to the collector. The delayed solidification allows sufficient time for instabilities caused by surface tension to fully contract the elongated jet into spherical microparticles prior to deposition. At later aging times, although the viscosity of the P­(PDC-BiOx) solution becomes comparable to that of the PVA solution, the rheological character is fundamentally different. The aged P­(PDC-BiOx) solution deviates upward from the Cox–Merz rule, indicating network formation. This behavior is unfavorable for electrospinning to produce fibers, as it does not permit smooth jet elongation under strong extensional and shear flows.

### 3.5 Biodegradation Behavior in Marine Environments

The biodegradability of P­(PDC-BiOx) in marine environments was evaluated by measuring the biochemical oxygen demand (BOD) in both surface and deep seawater at 25 °C for 45 days (Figure 7). The biodegradability (%) was estimated as the ratio of BOD consumption to the theoretical oxygen demand (ThOD). Cellulose powder was used as a positive reference material to confirm the biological activity of the microbial communities in the collected seawater samples. As shown in Figure 7, cellulose powder biodegraded rapidly, reaching over 90% in surface seawater within 20 days (Figure 7a) and approximately 80% in deep seawater after 45 days (Figure 7b). Although the cellulose filter paper exhibited almost the same level of biodegradation of 90% in surface seawater, it showed much lower biodegradation of approximately 40% in deep seawater. The lower biodegradation rate in deep seawater is likely related to lower microbial biomass or nutrient limitations.

**Figure 7:**
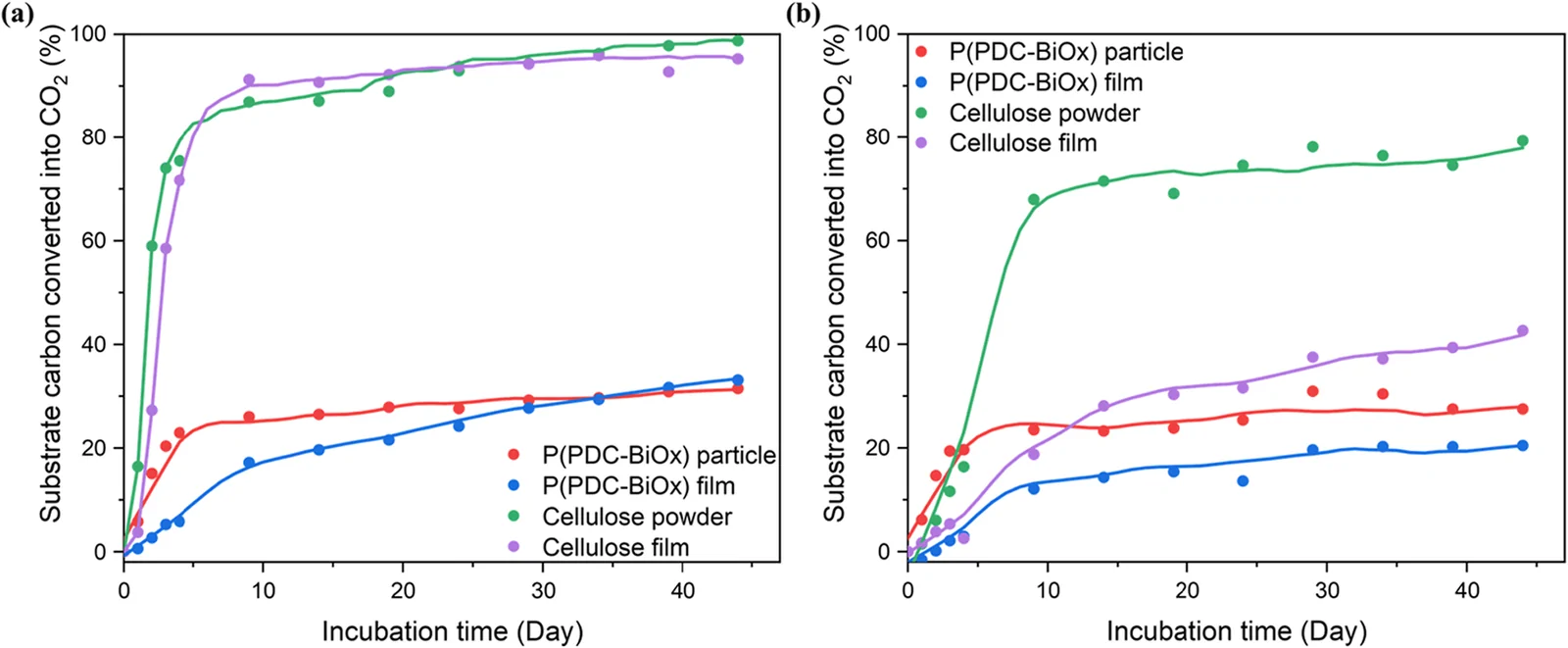
Marine biodegradation profiles of P­(PDC-BiOx) in the forms of hot-pressed films and electrosprayed microparticles compared with cellulose powder and filter paper for 45 days in (a) surface seawater and (b) deep seawater at 397 m depth. Experiments were conducted in accordance with ISO 23977-2:2020 (initiated on July 13, 2025). Average plots of three test samples.

It was found that the physical morphology of the polymer samples significantly influenced the biodegradation kinetics. In deep seawater, the electrosprayed P­(PDC-BiOx) microparticles exhibited a higher biodegradation rate than the hot-pressed film, reaching approximately 28% after 45 days compared to 20% for the film (Figure 7b). Consistent with the BET analysis (Figure S5), the specific surface area of the particles increased by approximately 15-fold compared to the dense film. While the absolute value (6.14 m^2^/g) is relatively modest compared to that of highly porous networks, for a solid thermoplastic polymer this transition from a flat geometry to discrete microparticles represents a substantial increase in the bio-accessible external surface area. This highly exposed macroscopic surface provided significantly more accessible docking sites for bulky marine bacteria and their secreted enzymes to initiate colonization and degradation, thereby accelerating the overall biodegradation process. In surface seawater, the effect of the surface area was most pronounced during the initial stage. The electrosprayed particles showed a rapid increase in biodegradation, reaching ∼20% within the first 4 days, whereas the film reached the same level only after 15 days (Figure 7a). Although the final biodegradability for both morphologies converged at approximately 30–33% after 45 days, the accelerated initial kinetics confirm that increasing the surface-to-volume ratio effectively promotes the onset of biodegradation.

To visually correlate these findings, the physical disintegration of the P­(PDC-BiOx) film was further evidenced by SEM observation after 7 days of immersion in surface seawater (Figure 3c). While the pristine film initially possessed a smooth and compact surface (Figure 3a), the specimen retrieved at day 7 exhibited significant morphological deterioration, characterized by the formation of deep pits, cracks, and interconnected voids. This rapid structural breakdown at the early stage is consistent with the initial burst of BOD profiles in Figure 7. Notably, the film became too fragile to be retrieved for SEM analysis after 15 days and eventually achieved complete macroscopic disappearance within the 45-day test period. These visual observations, combined with the quantitative BOD data, confirm that the marine microorganisms effectively colonized the amorphous P­(PDC-BiOx) surface and initiated rapid enzymatic erosion, leading to the total collapse of the polymer framework.

Overall, both hot-pressed film and microparticle forms of P­(PDC-BiOx) demonstrated clear biodegradability in surface seawater, achieving approximately 30% biodegradation within 45 days. To better contextualize this performance, the biodegradability of P­(PDC-BiOx) was compared with that of PHBV, a well-established marine-degradable poly­(hydroxyalkanoate) (PHA). Although PHBV achieved near-complete mineralization within 20 days (Figure S6), the ∼30% mineralization of P­(PDC-BiOx) remains highly promising for a synthetic polymer, given the substantial steric hindrance and strong intermolecular cohesion imposed by its rigid pseudo-aromatic rings and hydrogen-bonding amide groups. Considering that PDC is a biometabolic intermediate derived from lignin, its presence in the polymer backbone likely facilitates microbial recognition and metabolism.^24^ It should be noted that PDC itself showed approximately 80% of biodegradability in pond water.^32^ The substantial CO_2_ evolution confirmed by BOD measurements indicates that marine microorganisms possess the necessary enzymes to cleave the chemical bonds of P­(PDC-BiOx). Importantly, the absence of low-molecular-weight oligomers in the pristine polymer, as verified by the monomodal GPC trace (Figure S1), ensures that the measured oxygen consumption reflects true mineralization of the polymer backbone rather than the depletion of small-molecular impurities. The abrupt decrease in the biodegradation rate observed after 15 days in both environments suggests that the process is initially driven by the rapid erosion of highly accessible amorphous regions, followed by a transition into a plateau phase potentially limited by the bioavailability of the remaining intermediates.

## 4 Conclusions

In this study, a novel bio-based polyesteramide, P­(PDC-BiOx), was successfully synthesized via atom-economic ring-opening polyaddition. This synthetic strategy demonstrates an efficient approach for constructing lignin-derived polymer frameworks without the formation of small-molecular by-products. Although many studies on PDC have been reported, this is the first example of synthesizing a soluble linear polymer via direct polyaddition of PDC. Thermal and X-ray diffraction analyses confirmed that the resulting polymer exhibited completely amorphous characteristics. Due to the rigid heterocyclic structure of the PDC units in the backbone, the polymer films displayed high stiffness and a glassy state at ambient temperatures, while being characterized by brittle fracture behavior.

Notably, the biodegradation performance of P­(PDC-BiOx) in marine environments was found to be highly dependent on its physical morphology. While hot-pressed films showed limited accessibility for microbial interaction, transforming them into discrete microparticles via electrospraying significantly increased the specific surface area. This morphological change led to a pronounced acceleration in the initial biodegradation kinetics in both surface and deep seawater environments. These findings highlight the great potential of P­(PDC-BiOx) as a sustainable bio-based material. Furthermore, this study demonstrates that by strategically controlling the material morphology, the environmental lifespan of bio-based polymers can be effectively tuned, thereby offering broad prospects for functional applications. However, given that biodegradation reaches approximately 30% within 45 days, the initial physical fragmentation inevitably produces transient microplastics prior to complete mineralization, underscoring the need for extended environmental assessments in future studies.

## Supplementary Material



## Notes

Proposed reaction mechanism, optimization of reaction conditions, GPC chromatograms, DSC curve, tensile stress-strain curve, AFM images, nitrogen adsorption-desorption isotherms, marine biodegradation profiles, and NMR spectra (PDF)
