# Supplementary material for: Atom-Economic Synthesis and Morphology-Dependent Marine Biodegradation Behavior of 2‑Pyrone-4,6-Dicarboxylic Acid–Based Polyesteramide
Source: Polym Sci Technol. 2026 May 12;2(8):587–96. doi: 10.1021/polymscitech.6c00037 (PMC13520750; doi:10.1021/polymscitech.6c00037)
Supplement: Supplementary file 1 [file ps6c00037_si_001.pdf]

Supporting Information

Atom-Economic Synthesis and Morphology-Dependent Marine Biodegradation Behavior of 2-Pyrone-4,6-Dicarboxylic Acid-based Polyesteramide

Yijie Jin,[a] Wei Xin Tan,[a,b] Manjusha Joshi,[a] Yasutaka Kuwahara,[c] Shota Michida,[d] Atsushi Isobe,[a] Manabu Tanaka,[c] Takuya Katashima,[d] Masaya Fujita,[e] Takuma Araki,[f] Yuzo Suzuki,[f] Yuichiro Otsuka,[f] Naofumi Kamimura,[e] Eiji Masai,[e] and Tsuyoshi Michinobu[a,*]

[a] Department of Materials Science and Engineering, Institute of Science Tokyo, 2-12-1 Ookayama, Meguro-ku, Tokyo 152-8552, Japan.

[b] School of Materials Science & Engineering, Nanyang Technological University, Singapore 639798, Singapore.

[c] Department of Applied Chemistry, Faculty of Urban Environmental Sciences, Tokyo Metropolitan University, 1-1 Minami-osawa, Hachioji, Tokyo 192-0397, Japan.

[d] Department of Bioengineering, Graduate School of Engineering, The University of Tokyo, Tokyo 113-8656, Japan.

[e] Department of Materials Science and Bioengineering, Nagaoka University of Technology, Nagaoka 940-2188, Japan.

[f] Department of Forest Resource Chemistry, Forestry and Forest Products Research Institute, Tsukuba 305-8687, Japan.

E-mail: michinobu@mct.isct.ac.jp

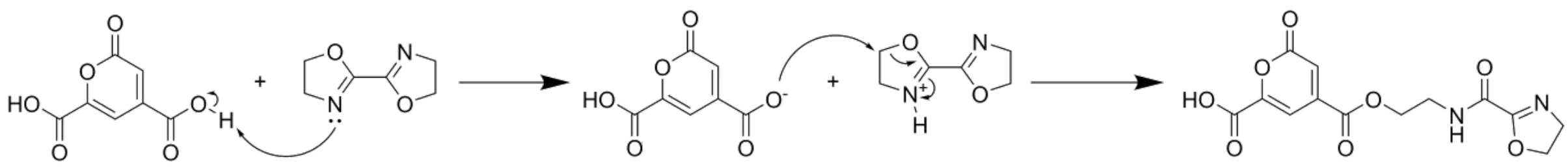

Scheme S1. Proposed mechanism for the ring-opening addition between the carboxylic acid group of PDC and the oxazoline ring.

Table S1 Optimization of reaction conditions for the synthesis of P(PDC-BiOx)

Reaction temp. (°C)	Reaction time (h)	M_n	M_w	PDI	Polymer formed	Yield (%)
80	18	4300	11000	2.50	Off-white solid	74
100	18	7400	15700	2.11	Off-white solid	74
120	3	11700	20200	1.74	Off-white solid	74
150	3	-	-	-	Brown hard precipitate	-

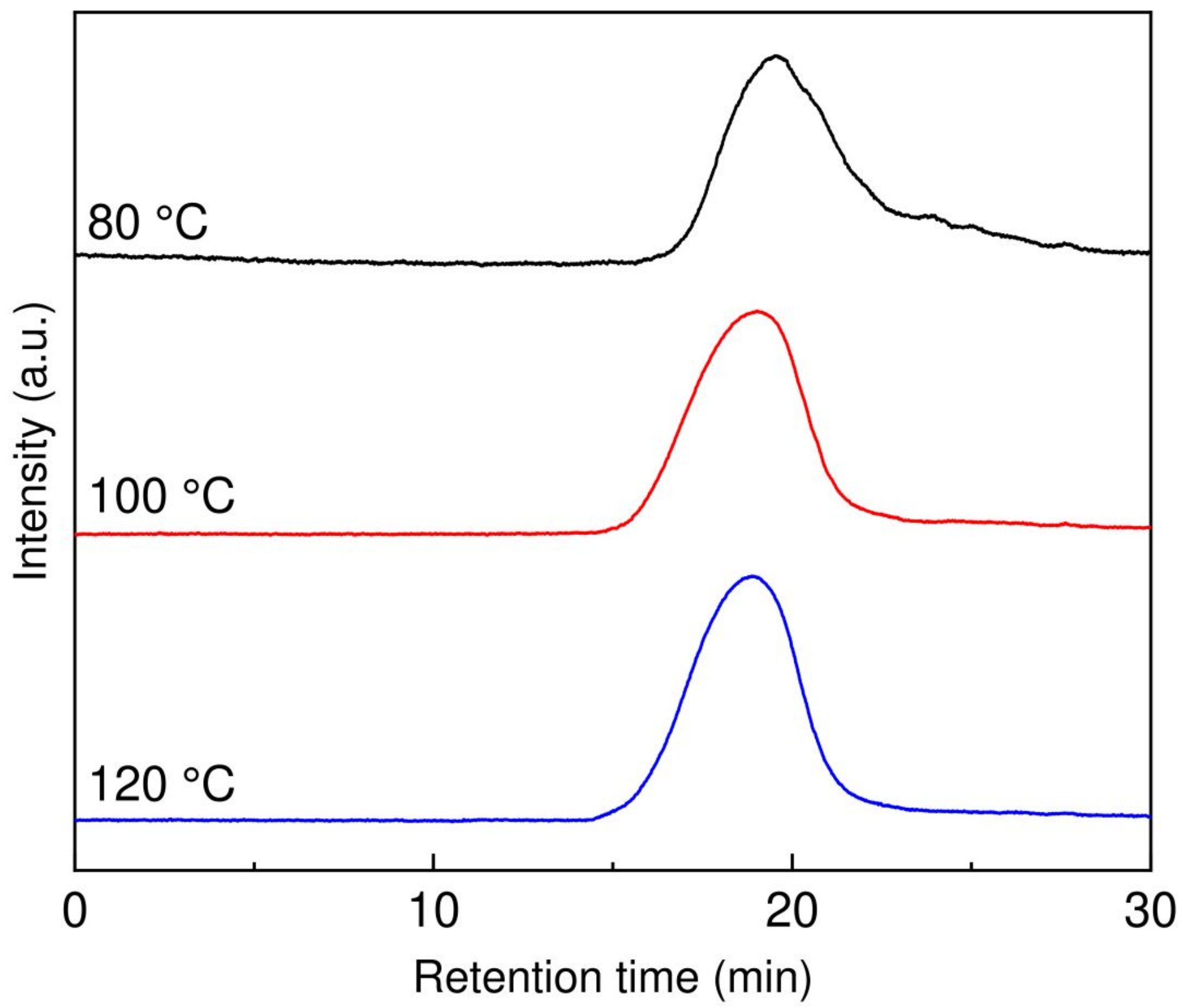

Figure S1. GPC chromatograms of P(PDC-BiOx) synthesized at different temperatures.

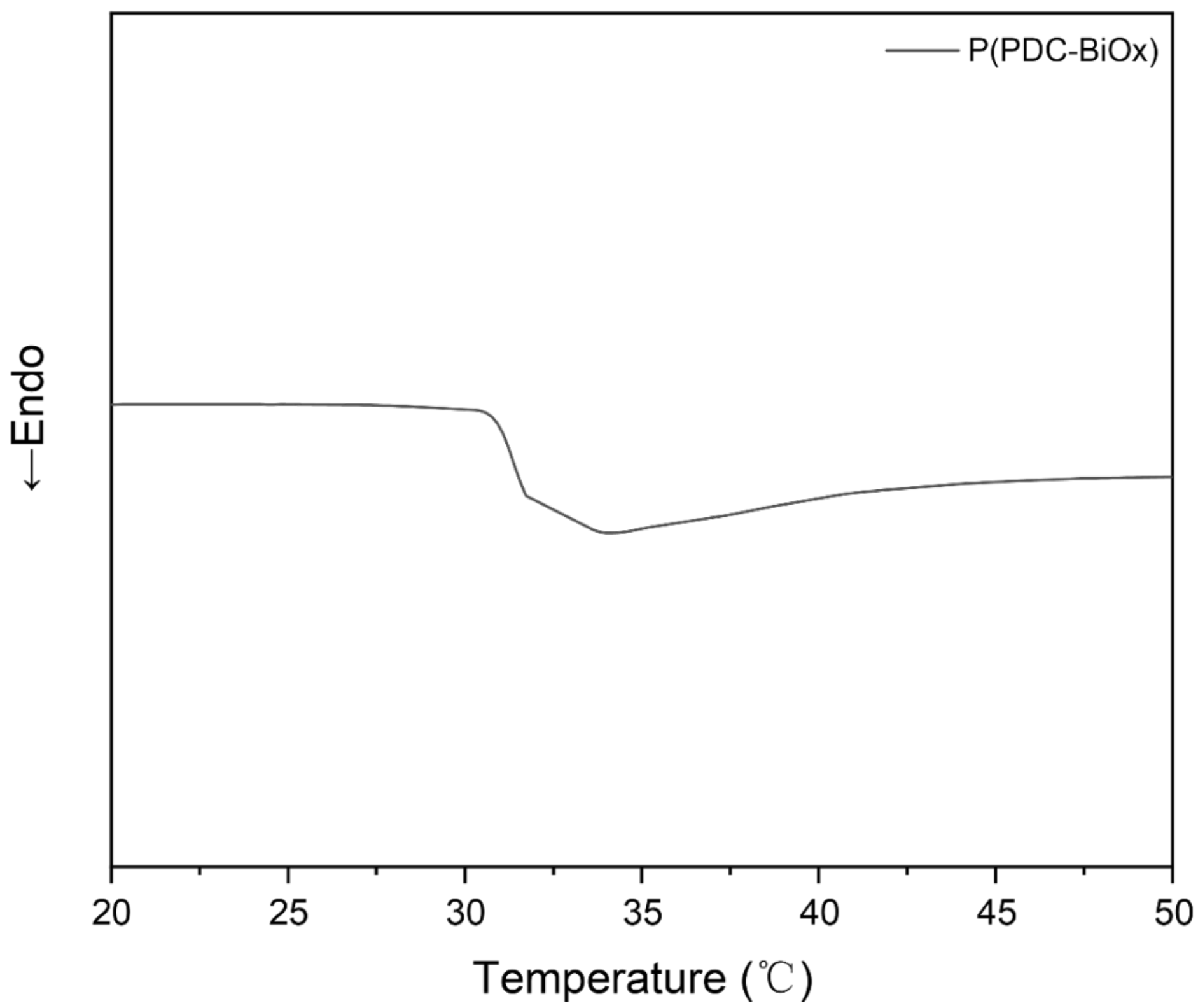

Figure S2. DSC curve (2nd heating scan) of P(PDC-BiOx) at the scanning rate of 10 °C min^{-1} under flowing nitrogen.

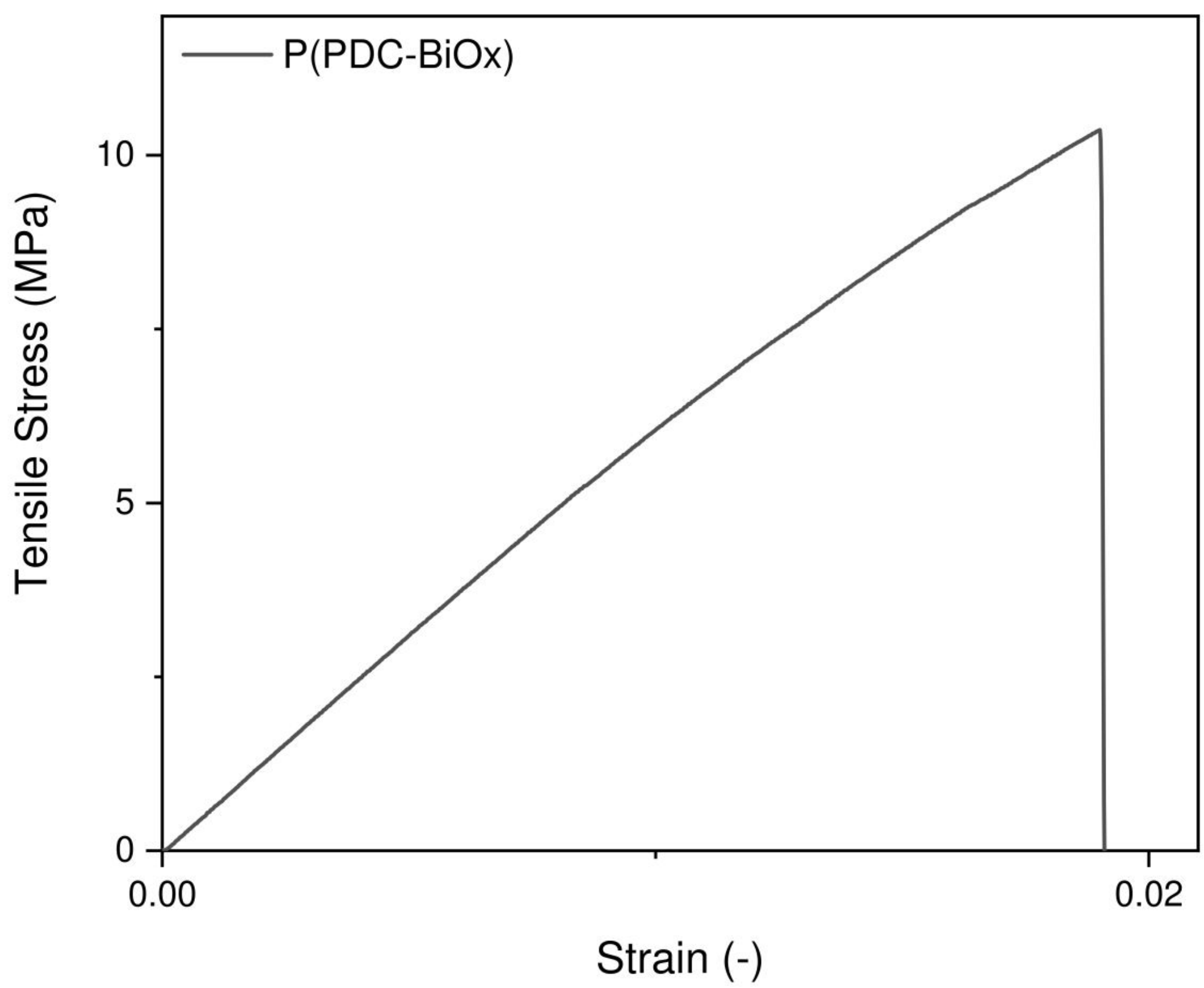

Figure S3. Tensile stress-strain curve of P(PDC-BiOx) hot-pressed film.

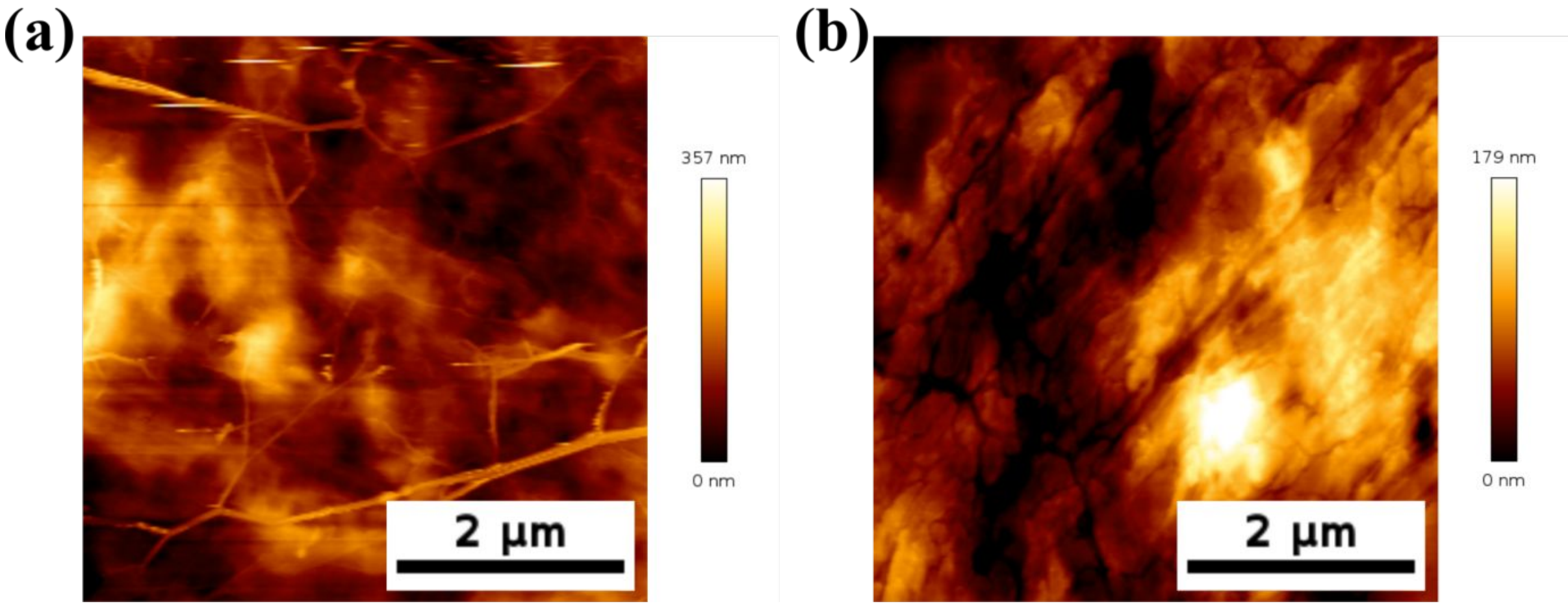

Figure S4. Atomic force microscopy (AFM) images of the P(PDC-BiOx) hot-pressed film (a) before and (b) after the marine biodegradation test.

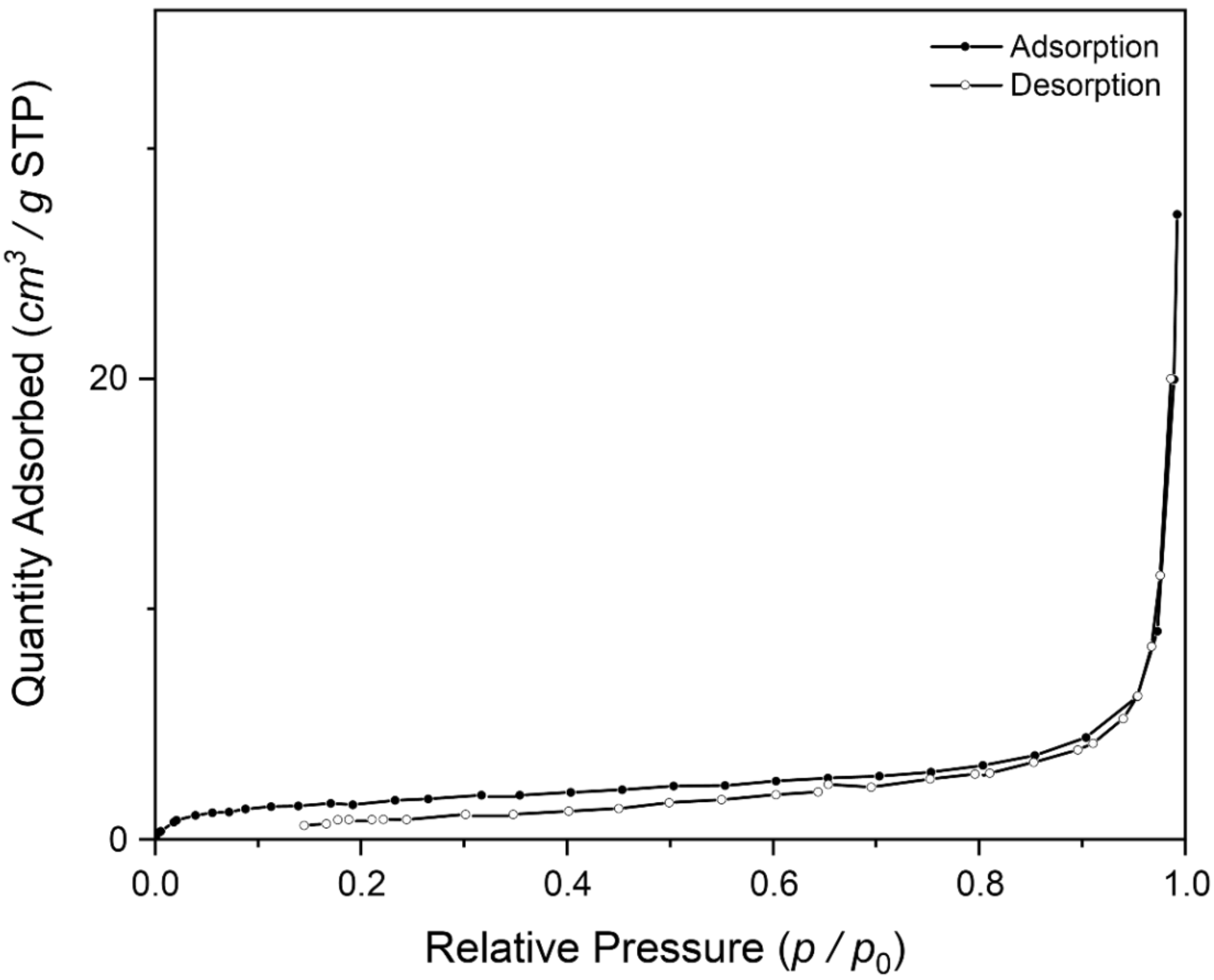

Figure S5. Nitrogen adsorption-desorption isotherms of the electrosprayed P(PDC-BiOx) microparticles.

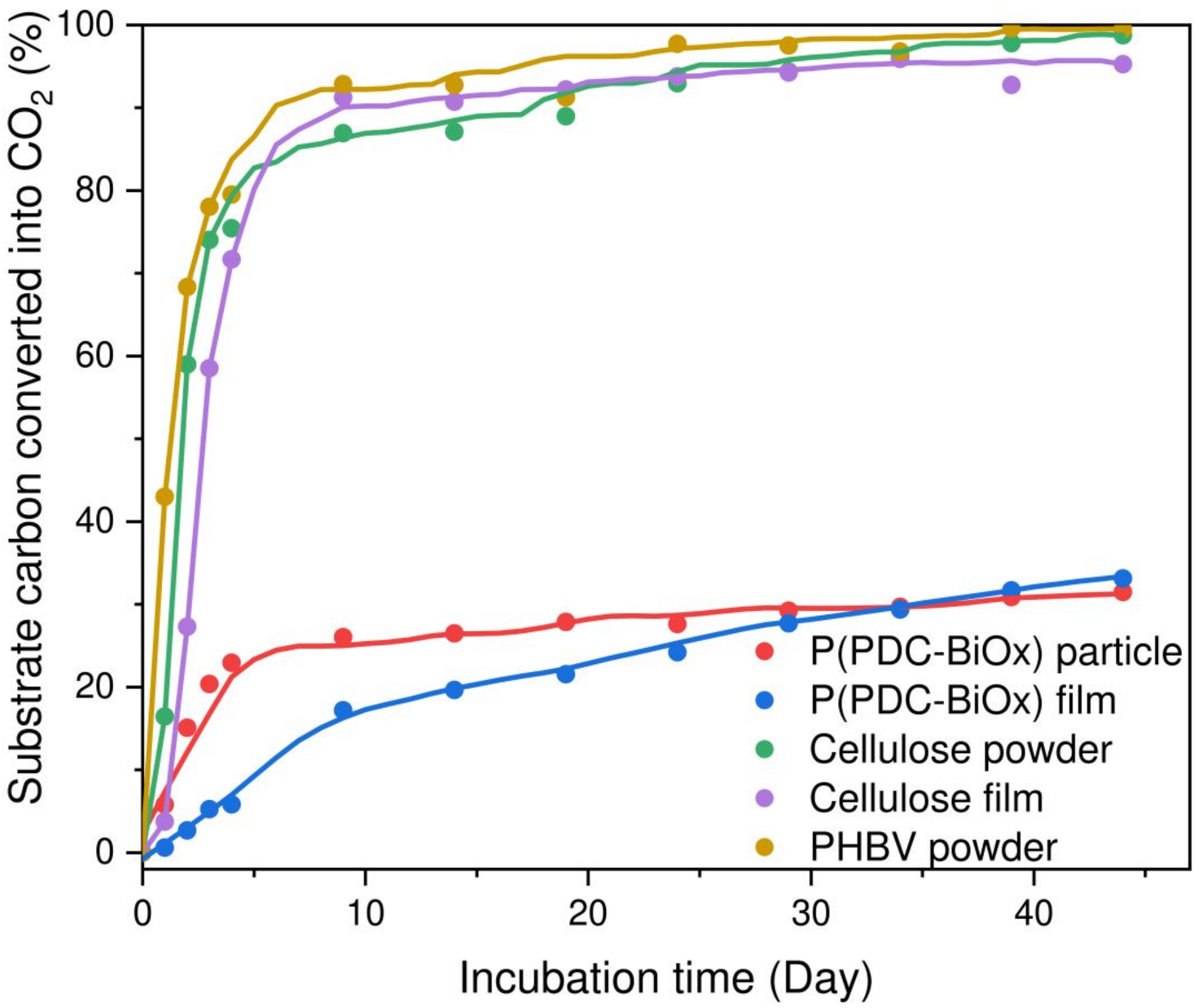

Figure S6. Marine biodegradation profiles of P(PDC-BiOx) compared with the PHBV reference in surface seawater.

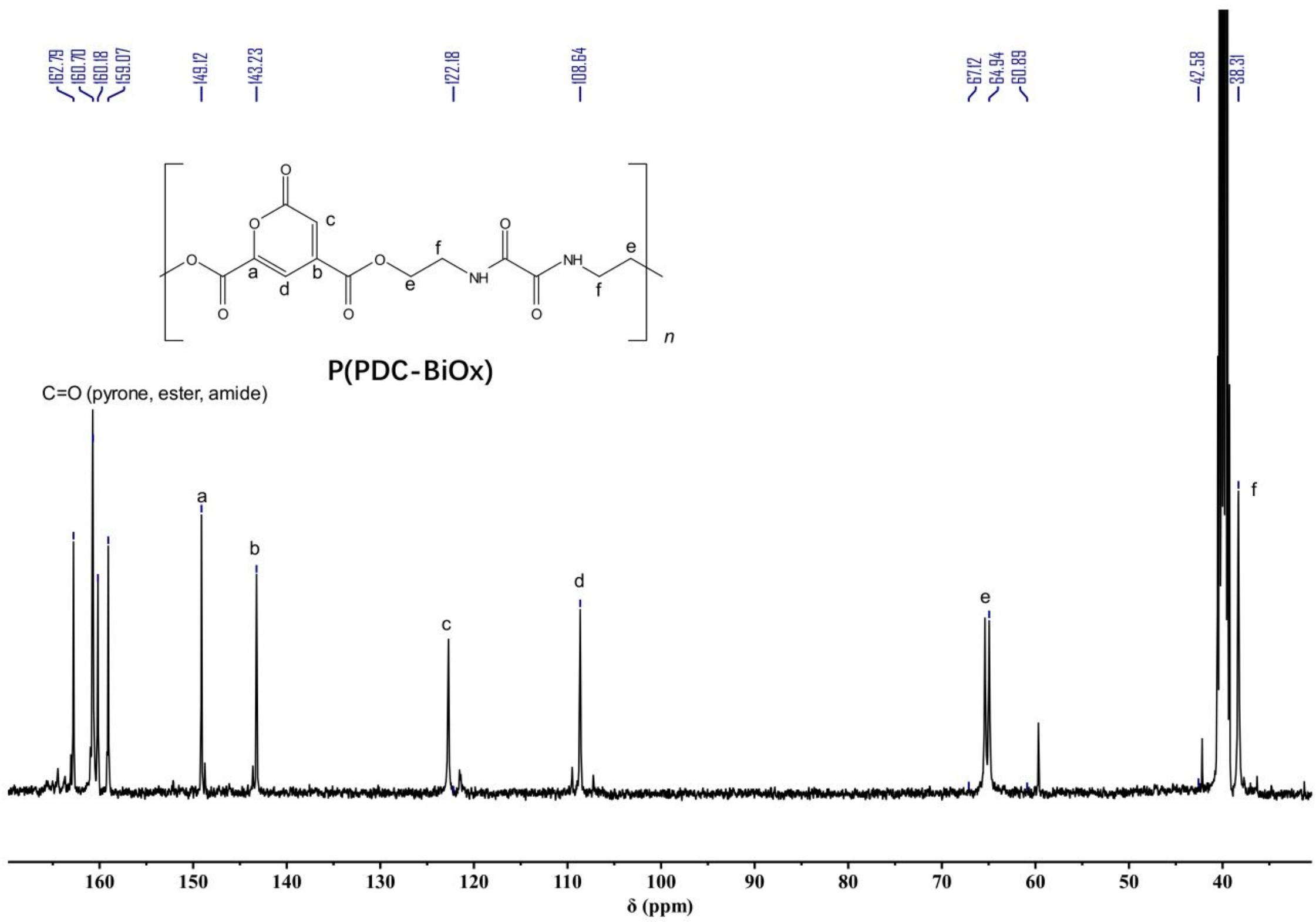

Figure S7. ^{13}C NMR spectrum of P(PDC-BiOx) in DMSO-d_6 at 20 °C

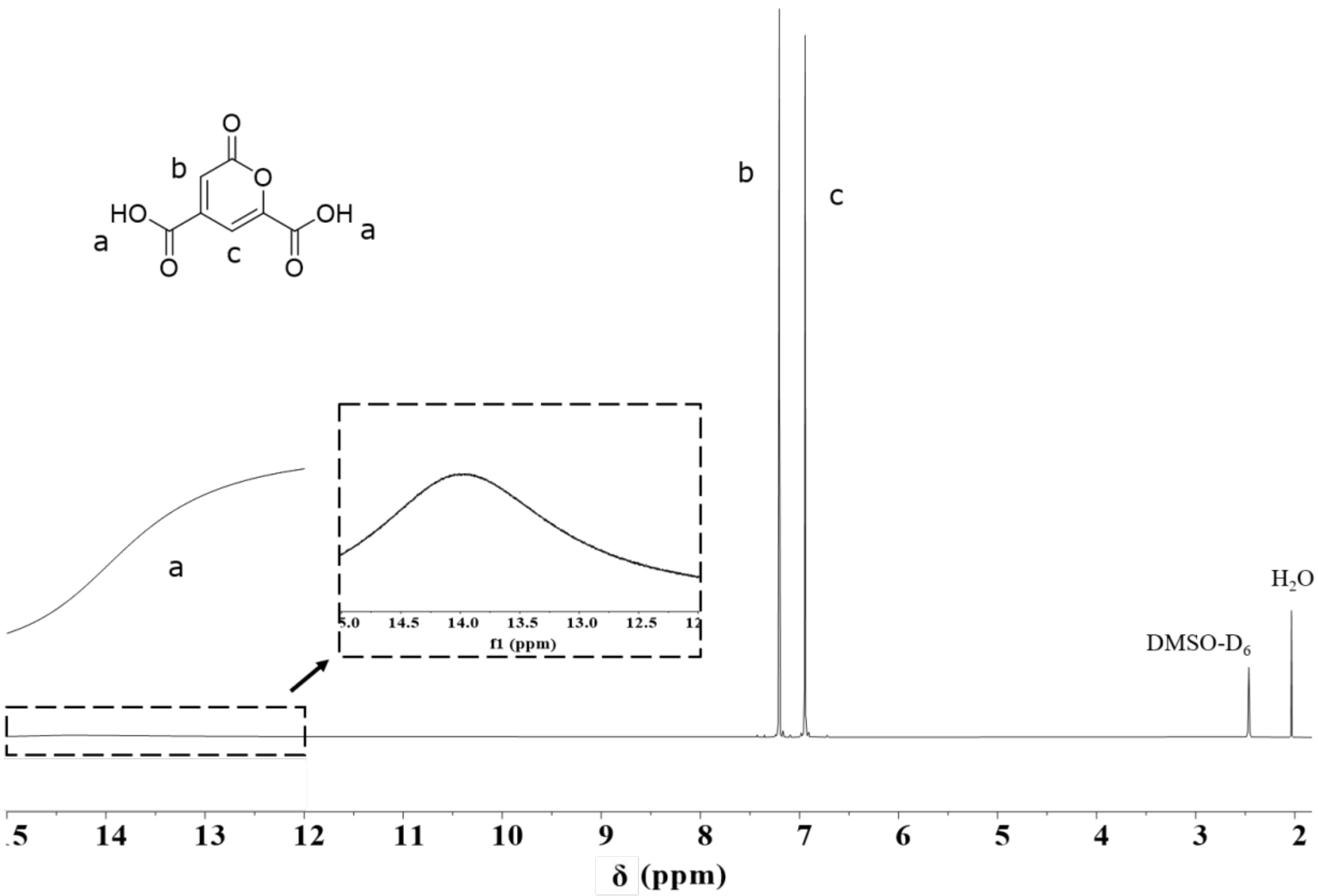

Figure S8. ^{1}H NMR spectrum of PDC in DMSO-d_6 at 20 °C
